# Case report: Depression in an older patient with dysgeusia as the initial symptom

**DOI:** 10.3389/fpsyt.2024.1478359

**Published:** 2024-12-10

**Authors:** Longrun Guo, Zhufa He

**Affiliations:** ^1^ Department of Rehabilitation Medicine, The Third People's Hospital of Ganzhou City, Ganzhou, Jiangxi, China; ^2^ Department of Psychiatry, The Third People's Hospital of Ganzhou City, Ganzhou, Jiangxi, China

**Keywords:** dysgeusia, depression, older, initial symptom, case report

## Abstract

**Introduction:**

Depression is the most common mental illness among older adults, with substantial and persistent mood depression as the main clinical feature, which is unfavorable for improvement. The clinical manifestations can range from melancholy to grief or even numbness. Approximately one-third of older adult patients exhibit physical discomfort as the first symptom; dysgeusia as the first symptom, is very rare in clinical practice. Dysgeusia is a clinical symptom with no specific objective indicator; thus, the likelihood of misdiagnosis and missed diagnosis is high.

**Case presentation:**

In this study, a 60-year-old female housewife with elementary school as her highest level of education, presented to the outpatient department with dysgeusia, poor sleep, and poor mood persisting for >1 year, which aggravated 2 weeks before hospital visit. Psychiatric examination showed clear consciousness; the patient was cleanly and timely dressed and demonstrated self-care but had a worrisome expression. Using the diagnostic criteria of the International Classification of Diseases (10^th^ edition), a diagnosis of a major depressive episode with psychotic symptoms was made. Following pharmacological and psychological treatment, the patient’s condition improved. The patient was compliant to treatment (10 mg/day of escitalopram), had a stable mood, good sleep, and no dysgeusia at >2 years of follow-up as an outpatient. She was able to do housework and take care of her children normally and did not complain about any other issues.

**Conclusion:**

Physical discomfort is a very common complaint in older patients with depression. If persistent physical symptoms do not improve after repeated medical treatment, timely assessment of the patient’s mental state or psychiatric referral should be considered to diagnose geriatric depression. Timely pharmacological and psychological therapy are the preferred treatment for older adults.

## 1 Introduction

Dysgeusia is a taste disorder that makes food taste metallic, bitter or sour or salty. It can manifest as conscious perception of the mouth being salty without eating salty food, or of having salty sputum or saliva (1, 2). Dysgeusia is typically characterized by an increase in sodium, potassium, calcium, and magnesium content in saliva, which is mostly caused by chronic pharyngitis, chronic nephritis, neurosis, oral ulcer, and heart failure (3–6). This article discusses the pathogenesis, diagnosis, differential diagnosis, and treatment of depression in older adults by reviewing literature and analyzing the diagnosis and treatment of a case of depression in an older patient with dysgeusia as the initial symptom. We aimed to deepen the clinical and pathological understanding of the disease and improve the recognition and treatment rate of depression in older adults. Here, we describe the digestive system as the first symptom of the identification of geriatric depression, thus reducing misdiagnosis and treatment.

## 2 Case description

The patient in this study was a 60-year-old female housewife, with elementary school as her highest level of education, who presented to the outpatient department on January 16, 2022, with dysgeusia, poor sleep, and poor mood persisting for >1 year. These complaints worsened over the 2 weeks preceding her hospital visit. In October 2020, the patient experienced persistent hiccups, a rush of gas inside the body, a persistent dysgeusia not reduced by eating sweet food, poor appetite, and a depressed mood. After the patient was examined in several hospitals in the city, brain magnetic resonance imaging, oral computed tomography, laryngoscopy, blood testing, liver and kidney function tests, and other examinations were performed, and no notable abnormalities were found. Treatment with digestive tablets, domperidone, and omeprazole was ineffective. During this period, the patient felt tired and weak, had a decreased ability to work, and reported that she told people around her repeatedly about the dysgeusia. The patient also had difficulty sleeping and repeatedly asked her family to take her to the hospital to resolve the dysgeusia. Two weeks before the hospital visit, the patient said that she felt bitterness in her heart and had repeated crying episodes. She also felt upset and very pessimistic and was worried that she might have cancer. Additionally, she had occasional suicidal ideations, did not feel able to take care of her children, was resting at home, and experienced notable weight loss (from 61 kg before the illness to 52 kg).

## 3 Diagnostic assessment

The patient had no previous history of a similar presentation. She has an introverted but strong personality with traits of a pursuit of perfection. The patient denied a family history of mental illness in the two previous generations. She had stable vital signs. Physical examination of the heart, lungs, and abdomen were normal. Psychiatric examination showed clear consciousness; the patient was cleanly and timely dressed and demonstrated self-care behaviors; however, she had an expression of worry. The patient took the initiative to communicate the dysgeusia, especially on the right side of the mouth, and temporary improvement of the dysgeusia upon eating sweet food. She felt like she had excess saliva but could not spit it out. The patient gave straight-to-the-point answers to questions, had a calm voice and good orientation, but showed signs of hypochondria, depression, and anxiety. She also experienced loss of energy, memory, appetite, and weight; hopelessness; suicidal ideations; sleep disturbance; and lack of self-awareness. Examination revealed taste disturbances. Auxiliary examination included liver and kidney function tests, an electrolyte panel, adrenocorticotropin and serum cortisol levels, color ultrasound, thyroid function tests, and blood, urine, and bowel routine testing, which demonstrated no abnormalities.

Regarding psychological testing, the 32-item hypomania symptoms checklist revealed a total score of 6, indicating no previous hypomanic episodes. The Hamilton Depression Scale-24 (HAMD-24) score was 36, indicating major depression, and the Hamilton Anxiety Scale (HAMA) score was 17, indicating the presence of anxiety. The Coping Style Questionnaire score was 151 points, indicating an immature coping style.

Considering the patient history, psychological assessment findings, and psychiatric examination findings, a diagnosis of major depressive episode with psychotic symptoms, according to the International Classification of Diseases (10^th^ edition) diagnostic criteria, was made. In terms of treatment, escitalopram 5 mg/day was administered initially and gradually increased to 10 mg/day. Alprazolam 0.4 mg/night was also prescribed. Simultaneously, psychological treatment was started, and the relationship between physical discomfort such as dysgeusia and bad mood was explained to the patient. Psychological education was also given to the family to improve their understanding and support. Two weeks later, the patient returned to the clinic and reported that her sleep had significantly improved, the dysgeusia decreased, her mood was less irritable, her diet had improved, and the HAMD-24 and HAMA scores were 27 and 10 points, respectively. Four weeks later, the symptoms had improved significantly; the dysgeusia was mostly absent, sleep was normal, mood and appetite had improved, and the patient could do housework and take care of her children. At this time, the HAMD-24 and HAMA scores were 14 and 7, respectively. Alprazolam was gradually discontinued, while escitalopram was continued at a daily dose of 10 mg. By the 8th and 16th week of treatment, the HAMD-24 scores had reduced to 8 and 5, respectively, indicating complete remission of clinical symptoms. The patient has been followed up in the outpatient department for over 2 years, adheres to medication, maintains a stable mood, and has good sleep quality. There have been no further complaints of a dysgeusia, and the patient has been able to manage household chores and care for her children. The patient continues to take escitalopram at 10 mg/day without reporting any other discomfort.

## 4 Discussion

Depression is the most common mental illness in older adults. Geriatric depression usually refers to depression in adults over 60 years old, including the first onset of depression in old age, depression with onset at a young age progressing or relapsing at an older age, and a variety of secondary depression disorders in older age, with a longer disease course. Depression has a tendency for remission and relapse; some cases have a poor prognosis and may progress to refractory depression (7–9). Different causes or types of geriatric depression have their own characteristics, such as cognitive function impairment. Some patients have physical discomfort or hypochondria as the main complaint. About one-third of patients over 60 years old experience physical discomfort as the first symptom, often involving the digestive system and presenting as belching, acid reflux, and bloating (10–12). The patient in this study had dysgeusia as the first symptom, which is very rare in clinical practice. In the absence of salty food, dysgeusia can be considered a phantom taste symptom. Some older patients are prone to develop hypochondria and report physical discomfort when depression is serious and can even progress to hypochondriac delusion (13, 14).

Dysgeusia not triggered by salty food in the mouth is a type of taste disturbance. Common causes of taste disturbances include abnormalities in the taste conduction process, olfactory disorders, systemic diseases and related treatment history, and other chronic diseases and related treatment histories. These include Parkinson’s disease, viral infections, diabetes, endocrine disorders, chronic bronchitis, malnutrition, pernicious anemia, hemodialysis, and the use of certain medications, such as macrolides and tetracyclines, tricyclic antidepressants, anti-anxiety drugs, thyroid regulators, anti-cancer drugs, anti-inflammatory drugs, and antihistamines. Psychological factors can trigger and exacerbate taste disturbances. In this case, while experiencing dysgeusia, the patient visited the oral department of a general hospital, where a history of oral diseases was ruled out. The patient had no history of other diseases and had not used any related medications; thus, taste disturbances caused by the above factors were ruled out.

In our case, the patient was an older woman with a disease course of >1 year, which may have been due to poor adaptation to a new living environment. The patient gradually developed persistent hiccups, dysgeusia, fatigue, sleep disorder, anxiety, and depression, and significantly impaired social function. The patient had visited multiple hospitals in the city repeatedly for dysgeusia, and the clinicians did not know enough about the patient’s diet, sleep, and mood. Additionally, the dysgeusia symptoms are mainly clinical and subjectively reported by patients, without specific objective indicators, which increases the likelihood of misdiagnosis and missed diagnosis. Therefore, especially in older patients with depression, persistent physical symptoms, and lack of improvement after repeated medical treatment, timely assessment of the patient’s mental state or psychiatric referral is crucial for timely diagnosis and treatment. Fortunately, after referral to the psychology department, the patient was able to get timely treatment.

In our case, selective serotonin reuptake inhibitors were used as antidepressants. Although the patient’s phantom symptoms were considered, antipsychotic medication was not given. In the short term, the combination of benzodiazepines and anti-anxiety treatment improved the patient’s anxiety symptoms and sleep, and the dysgeusia did not recur. In the treatment of geriatric depression, it is advisable to start with a small dose, perform individualized titration, and attempt to use monotherapy to reduce the interaction between drugs and improve medication compliance. Concurrently, it is also critical to strengthen treatment with psychological interventions, pay attention to disease health education, and establish a good doctor–patient relationship and family support system for the treatment and rehabilitation of these patients.

Geriatric depression often takes various physical symptoms as the first symptom, but the manifestation of dysgeusia symptoms, which can easily be misdiagnosed as oral disease, is rare. We report the first case of geriatric depression with dysgeusia as the first symptom, which provides clinical experience for diagnosis and treatment.

## 5 Patient perspective

The patient agrees with the diagnosis and treatment of the disease and has signed an informed consent form.

## Data Availability

The original contributions presented in the study are included in the article/supplementary material. Further inquiries can be directed to the corresponding author.

## References

[1] Chen X Luo N Guo C Luo J Wei J Zhang N . Current trends and perspectives on salty and salt taste-enhancing peptides: A focus on preparation, evaluation and perception mechanisms of salt taste. Food Res Int. (2024) 190:114593. doi: 10.1016/j.foodres.2024.114593 38945609

[2] Taruno A Gordon MD . Molecular and cellular mechanisms of salt taste. Annu Rev Physiol. (2023) 85:25–45. doi: 10.1146/annurev-physiol-031522-075853 36332657

[3] Bigiani A . Salt taste, nutrition, and health. Nutrients. (2020) 12:1537. doi: 10.3390/nu12051537 32466165 PMC7284473

[4] Tan SY Tuli P Thio G Noel B Marshall B Yu Z . A systematic review of salt taste function and perception impairments in adults with chronic kidney disease. Int J Environ Res Public Health. (2022) 19:12632. doi: 10.3390/ijerph191912632 36231932 PMC9564527

[5] Wu Y Liao F Liao L Li F Yang Y Fan M . Salt taste threshold and contributory factors of chronic kidney disease patients: A cross-sectional study. Int Urol Nephrol. (2023) 55:1211–8. doi: 10.1007/s11255-022-03403-1 36318407

[6] Cohen LP Wessler JD Maurer MS Hummel SL . Salt taste sensitivity and heart failure outcomes following heart failure hospitalization. Am J Cardiol. (2020) 127:58–63. doi: 10.1016/j.amjcard.2020.04.008 32416964 PMC7452726

[7] Casey DA . Depression in older adults: A treatable medical condition. Prim Care. (2017) 44:499–510. doi: 10.1016/j.pop.2017.04.007 28797375

[8] Almeida OP . Prevention of depression in older age. Maturitas. (2014) 79:136–41. doi: 10.1016/j.maturitas.2014.03.005 24713453

[9] Steffens DC . Treatment-resistant depression in older adults. N Engl J Med. (2024) 390:630–9. doi: 10.1056/NEJMcp2305428 PMC10885705 38354142

[10] Zis P Daskalaki A Bountouni I Sykioti P Varrassi G Paladini A . Depression and chronic pain in the elderly: Links and management challenges. Clin Interv Aging. (2017) 12:709–20. doi: 10.2147/CIA.S113576 PMC5407450 28461745

[11] Jonsson U Bertilsson G Allard P Gyllensvärd H Söderlund A Tham A . Psychological treatment of depression in people aged 65 years and over: A systematic review of efficacy, safety, and cost-effectiveness. PLoS One. (2016) 11:e0160859. doi: 10.1371/journal.pone.0160859 27537217 PMC4990289

[12] Ni Y Tein JY Zhang M Yang Y Wu G . Changes in depression among older adults in China: A latent transition analysis. J Affect Disord. (2017) 209:3–9. doi: 10.1016/j.jad.2016.11.004 27866046

[13] MaChado L Filho LE MaChado L . When the patient believes that the organs are destroyed: Manifestation of Cotard’s syndrome. Case Rep Med. (2016) 2016:5101357. doi: 10.1155/2016/5101357 28003827 PMC5149641

[14] Ferraris C Scarlett CJ Bucher T Beckett EL . Liking of salt is associated with depression, anxiety, and stress. Chem Senses. (2023) 48:bjad038. doi: 10.1093/chemse/bjad038 37738157 PMC10628984

